# Recent progress in serine metabolism reprogramming in tumors and strategies for serine deprivation

**DOI:** 10.3389/fonc.2025.1669565

**Published:** 2025-12-03

**Authors:** Huimei Liu, Yanping Liu

**Affiliations:** 1 College of Life Science and Technology, Tarim University, Alar, Xinjiang, China; 2 State Key Laboratory Incubation Base for Conservation and Utilization of Bio-Resource in Tarim Basin, Alar, Xinjiang, China; 3 Department of Nursing, The First Affiliated Hospital of Chongqing Medical University, Chongqing, China

**Keywords:** metabolic reprogramming, serine synthesis pathway, phosphoglycerate dehydrogenase, serine deprivation, nanodelivery systems

## Abstract

Tumor cells undergo extensive metabolic reprogramming during malignant proliferation, with serine—a key nonessential amino acid—playing multiple roles in tumor metabolism. To maintain high serine levels, tumor cells upregulate phosphoglycerate dehydrogenase to enhance endogenous synthesis and concurrently increase exogenous uptake. Serine deprivation demonstrates antitumor potential across various malignancies; however, its clinical application remains limited by inadequate tumor selectivity and systemic toxicity. Recent advances in nanodelivery systems offer precise strategies to modulate tumor serine metabolism. Serine deprivation via these systems improves tumor-specific targeting while minimizing off-target toxicity to normal tissues. Therefore, this review aims to outline serine metabolism and its regulatory networks, evaluate the therapeutic potential and limitations of serine deprivation, and highlight recent advances in nanodelivery strategies targeting serine metabolism for cancer therapy, thereby providing insights for the development of novel anticancer approaches.

## 1 Introduction

Tumor cell proliferation and survival depend on their unique metabolic reprogramming, characterized by altered energy metabolism and elevated demand for biomolecules such as amino acids, nucleotides, and lipids (1, 2). Advances in tumor metabolism highlight the regulatory mechanisms of metabolic reprogramming—recognized as a hallmark of malignant tumors (3, 4)—as a pivotal focus in tumorigenesis and progression (5). Tumor cells reprogram their metabolic pathways to meet elevated energy demands, generate metabolic precursors essential for rapid proliferation, and supply substrates for macromolecule biosynthesis, including proteins (6), lipids (7), and nucleic acids (8, 9). Among these phenomena, the “Warburg effect” exemplifies tumor metabolic reprogramming, where glucose is preferentially converted to lactate under aerobic conditions instead of producing ATP efficiently via oxidative phosphorylation (10). This phenomenon is a hallmark of autonomous metabolic reprogramming across diverse malignant tumors. Its discovery has fundamentally advanced the field by initiating extensive investigations into the mechanisms of metabolic reprogramming in cancer (11). Beyond the glucose metabolic abnormalities of the Warburg effect, tumor cells exhibit additional metabolic dysregulation, particularly in lipid (12) and amino acid metabolism (13). Amino acids, the primary cellular nutrients and energy sources after glucose, serve as essential substrates for protein synthesis. Reprogrammed amino acid metabolism is central to tumorigenesis and cancer progression (14, 15).

Amino acid metabolic reprogramming involves coordinated alterations in uptake rates, metabolic pathways, key metabolic enzyme expression, and metabolite production within tumor cells during tumorigenesis and progression. These systemic modifications enable them to meet the demands of rapid proliferation and facilitate survival within the hostile tumor microenvironment (16, 17). Across diverse tumor types, amino acids—glutamine, serine, arginine, tryptophan, and aspartate—drive metabolic reprogramming and regulate key biological processes including proliferation, invasion, migration, and immune evasion (15, 18–20). Serine is the third most consumed nutrient by tumor cells, after glucose and glutamine, and represents the second-largest amino acid—associated metabolic reprogramming event after glutamine (21, 22). Although glutamine metabolism has been widely studied in the context of tumor metabolic reprogramming, serine metabolism is emerging as a key focus in cancer metabolism research (23, 24). Serine is a central node in one-carbon metabolism (25–27), acting as a substrate for protein synthesis (28), a methyl-group donor, and a phospholipid precursor that supports nucleotide biosynthesis and membrane biogenesis—processes essential for malignant cell proliferation (29, 30). Tumor cells enhance serine metabolism by upregulating the phosphoglycerate dehydrogenase (PHGDH)-mediated *de novo* synthesis pathway (31, 32) and activating exogenous transport systems (33).

Across diverse tumor models, inhibition of the serine synthesis pathway (SSP) or exogenous serine uptake suppresses proliferation, promotes immune infiltration, and inhibits metastasis, highlighting the blockade of serine metabolism as a promising target for antitumor therapy (34, 35). Nevertheless, conventional serine-deprivation strategies face several challenges, including limited tumor targeting specificity (36), off-target effects on serine uptake in normal cells (37), and tumor cell adaptation to evade therapeutic pressure through the activation of compensatory metabolic pathways, such as upregulated SSP activity (38). These limitations compromise the efficacy and safety of such therapies. Recent advances in the development of nanodelivery systems offer innovative solutions to address these challenges (39). Nanocarriers with active or passive targeting capabilities enable selective accumulation of therapeutics in the tumor microenvironment or specific tumor cells, thereby significantly enhancing antitumor efficacy while reducing off-target toxicity to normal tissues (40–42). This approach may enhance the antitumor efficacy of serine deprivation therapy and provide new insights and technical support for metabolism-based antitumor research.

Therefore, this review aims to summarize a comprehensive overview of tumor cell dependence on serine metabolism and its primary sources. After evaluating the therapeutic potential and limitations of serine-deprivation strategies in cancer treatment, the findings could guide the development of a nanodelivery-based approach for more targeted, effective, and safer antitumor treatments.

## 2 Sources of serine

The high metabolic demand for serine makes tumor cells dependent on extracellular serine uptake and on *de novo* production via the SSP (26, 27). Serine acquisition in the human body primarily occurs through the following pathways (1): uptake of extracellular serine via amino acid transporters such as SLC1A4 (43, 44), and (2) conversion of glycine to serine by SHMT1/2, facilitated by the glycine cleavage system (45). Although serine and glycine are interconvertible, extracellular glycine cannot substitute for serine in supporting tumor cell proliferation because the reverse conversion of glycine to serine consumes one-carbon units (45). Additionally, tumor cells can synthesize serine via the SSP, a key route for endogenous serine biosynthesis (34, 46). Studies show that over 80% of intracellular serine in tumor cells is produced through the SSP. Inhibition of the SSP impairs tumor cell proliferation, even in the presence of abundant extracellular serine or glycine (47). For example, in human breast cancer cells with *PHGDH* gene amplification, *PHGDH* knockout significantly reduced cell proliferation, an effect not rescued by excess serine supplementation in the culture medium (48, 49). Hence, the SSP is essential for tumor cell proliferation and survival.

**Figure 1:**
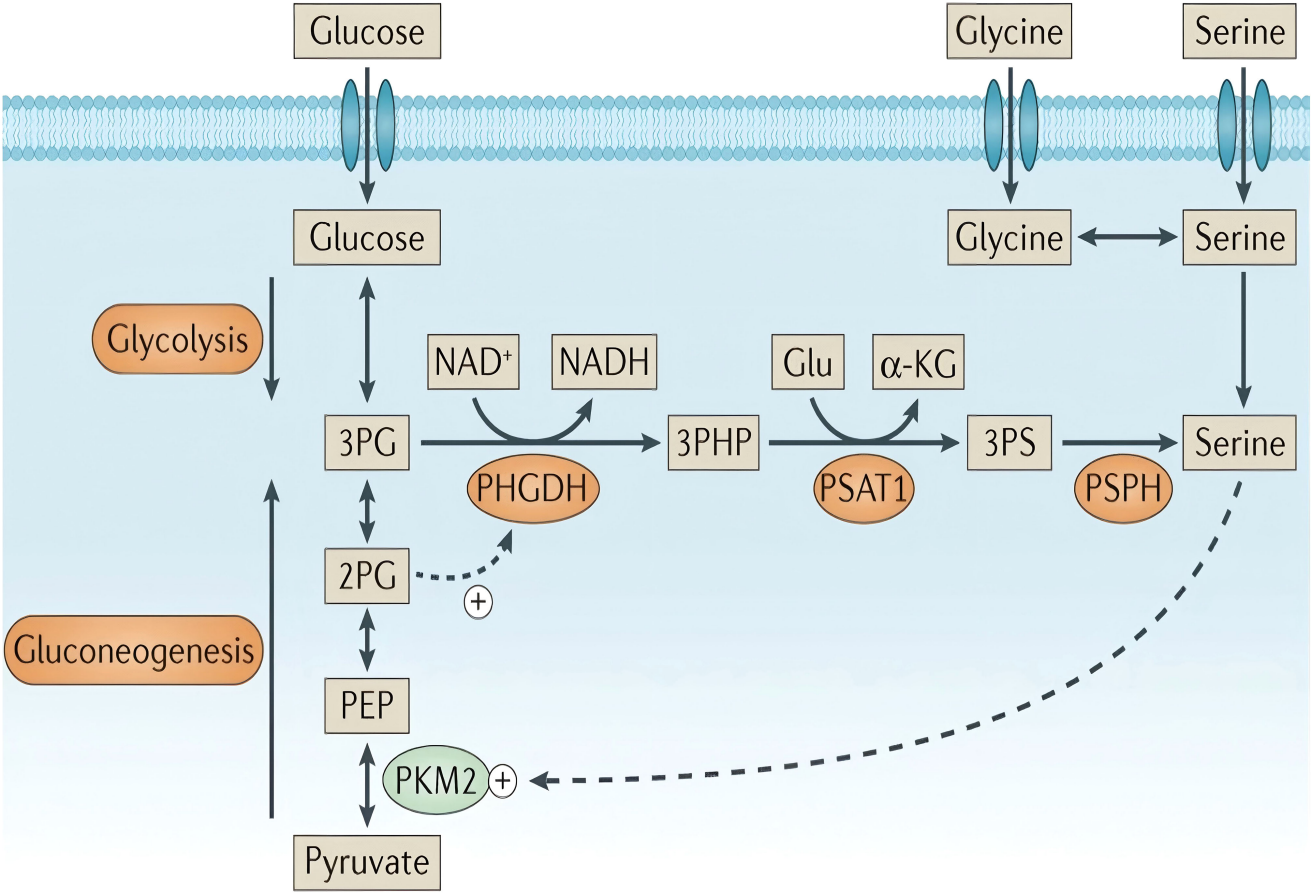
Serine synthesis pathway. “+” denotes activation, while dashed arrows indicate allosteric regulation. Copyright 2016, Springer Nature. α-KG, α-ketoglutarate; PEP, phosphoenolpyruvate.

In tumor cells, serine and glycine are obtained either through uptake by neutral amino acid transporters or *de novo* synthesis through the SSP. The *de novo* SSP, a key glycolytic branch exemplifying the Warburg effect (i.e., high glycolytic flux), is catalyzed by three enzymes (**Figure 1**, highlighted by an orange-yellow oval). In the first step, PHGDH catalyzes the oxidation of the glycolytic intermediate 3-PG to 3-phosphohydroxypyruvate (3-PHP), concurrently reducing NAD+ to NADH, and functions as the rate-limiting enzyme of the pathway. Additionally, 2-phosphoglycerate (2-PG) activates PHGDH (**Figure 1**, dashed line). Subsequently, phosphoserine aminotransferase (PSAT1) catalyzes the transamination of 3-PHP and glutamic acid to form 3-phosphoserine (3-PS) and α-ketoglutarate (α-KG). Finally, phosphoserine phosphatase (PSPH) dephosphorylates 3-PS to produce serine (50–52). Under serine-deprived conditions, reduced pyruvate kinase M2 activity redirects 3-PG flux into the SSP, promoting its compensatory activation.

In response to limited exogenous serine availability, tumor cells undergo metabolic reprogramming to upregulate the endogenous SSP, sustaining their survival and proliferation (38). During this adaptive response, the tumor suppressor gene p53 promotes *de novo* serine biosynthesis by transcriptionally activating key SSP enzymes (53). In melanoma (54) and breast cancer (55), PHGDH gene copy number is significantly increased, supporting the notion that tumor cells upregulate endogenous SSP activity in response to metabolic stress.

## 3 Key metabolic networks of serine

### 3.1 Serine and central carbon metabolism

**Figure 2:**
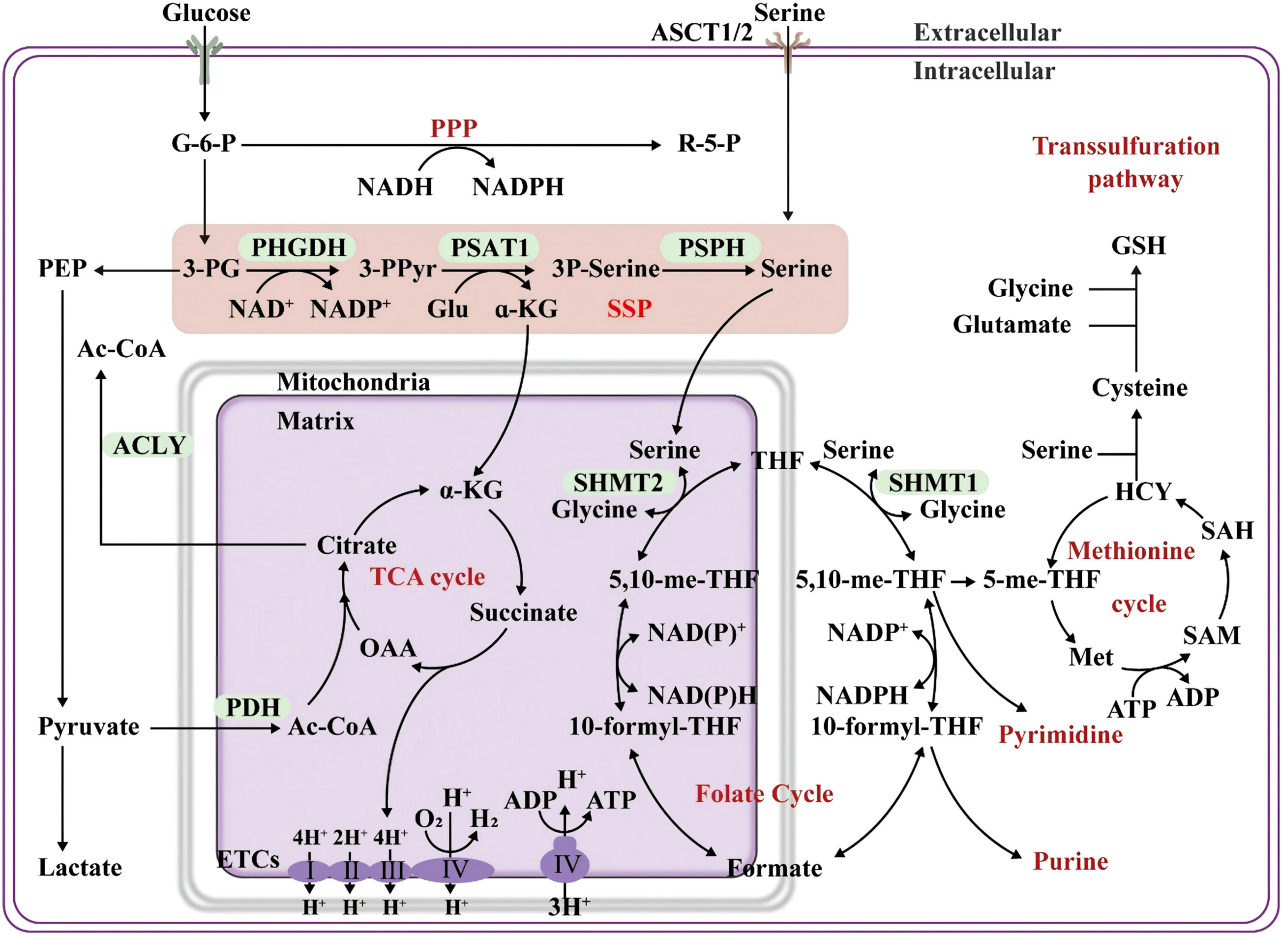
Overview of serine metabolism. Serine metabolism is closely linked to one-carbon metabolism, including the folate and methionine cycles, supporting the *de novo* synthesis of nucleic acid precursors (purines and pyrimidines), redox regulators (NADPH and GSH), and the methylation donor SAM. These metabolites are essential for cell proliferation, survival, differentiation, and epigenetic regulation. Copyright 2024, Springer Nature. SAM, S-adenosylmethionine.

The *de novo* SSP and the tricarboxylic acid (TCA) cycle share key intermediate metabolites, positioning serine metabolism as a central hub for tumor cell energy production and biosynthesis (**Figure 2**). SSP-derived α-KG serves as an anaplerotic substrate for the TCA cycle (55–57). Within the TCA cycle, α-KG is converted to oxaloacetate through sequential reactions, which then condense with acetyl-CoA to form citrate (**Figure 2**, mitochondrial TCA cycle). After cytosolic export, citrate is sequentially converted to acetyl-CoA by ATP-citrate lyase (ACLY), linking mitochondrial metabolism to the cytosolic *de novo* fatty acid synthesis pathway (58) (**Figure 2**, left-hand compartment of the cytosol). Furthermore, α-KG serves as an amino group acceptor for synthesizing nonessential amino acids, including glutamate, proline, and arginine. Therefore, serine metabolism is closely linked to the tumor cell biomass production and energy metabolism. Active SSP consumes substantial amounts of glutamate/glutamine, potentially competing with α-KG-producing other pathways, such as glutamate dehydrogenase. To maintain the TCA cycle, tumor cells often replenish α-KG via glutaminolysis. This indicates tumor metabolic plasticity: upregulated SSP drives enhanced glutamine metabolism, a phenomenon termed “glutamine addiction” (57).

### 3.2 Serine and one-carbon metabolism

Serine is the primary donor of one-carbon units and is essential for intracellular one-carbon metabolism. Cytosolic-synthesized serine is transported into the mitochondrial matrix by the mitochondrial transporter Sideroflexin 1 (SFXN1) (59). SFXN1 localizes to the inner mitochondrial membrane, with its N-terminus in the matrix and C-terminus in the intermembrane space. Upon entering the intermembrane space, serine is specifically recognized and bound by SFXN1 and transported into the mitochondrial matrix, initiating one-carbon metabolism (59). SFXN1 drives malignant tumor progression via canonical (metabolic) and noncanonical (non-metabolic) pathways. SFXN1 inhibition, mimicking mitochondrial serine deprivation, exerts anti-proliferative effects on tumors similar to those of dietary serine deprivation (60). In the mitochondria, serine hydroxymethyltransferase 2 catalyzes the transfer of the hydroxymethyl group of serine to tetrahydrofolate, producing glycine and 5,10-methylenetetrahydrofolate (5,10-CH_2_-THF) (**Figure 2**, folate cycle) (45, 61, 62). 5,10-CH_2_-THF, a one-carbon unit carrier, can be enzymatically converted into other one-carbon unit forms for diverse biosynthetic processes.

5,10-CH_2_-THF is exported into the cytosol as formate by methylenetetrahydrofolate dehydrogenase 2 and subsequently converted by methylenetetrahydrofolate dehydrogenase 1 into 10-formyltetrahydrofolate (**Figure 2**, right cytosolic compartment). This metabolite provides essential one_carbon units for purine and thymidylate synthesis, supporting DNA replication and cellular proliferation in tumor cells (63). 5,10-CH_2_-THF also participates in mitochondrial tRNA formylation, a critical step for mitochondrial protein synthesis (64). Therefore, serine one-carbon metabolism is upregulated in rapidly proliferating cancer cells to satisfy the high nucleotide demand.

Methylenetetrahydrofolate reductase reduces 5,10-CH_2_-THF to 5-methyl-THF. Subsequently, methionine synthase catalyzes the conversion of 5-methyl-THF and homocysteine to methionine (Met). Met adenosyltransferase adenosylates Met to produce the universal methyl donor S-adenosylmethionine (SAM) (45). Various methyltransferases convert SAM to S-adenosylhomocysteine (SAH), releasing methyl groups in the process. Ultimately, SAH is hydrolyzed to homocysteine, thereby completing the Met cycle (**Figure 2**). SAM serves as the principal methyl donor in most cellular methylation reactions (RNA, DNA, and histone methylation) (65, 66). SAM and SAH expression levels regulate methylation balance, a key factor in tumor epigenetic regulation (67, 68).

Serine metabolism is crucial for maintaining intracellular redox balance in tumor cells. Intracellular reactive oxygen species (ROS) critically regulate cellular homeostasis and function (69). ROS exert concentration-dependent effects on cellular fate: low levels promote proliferation, moderate levels induce genomic instability and mutagenesis, and excessive levels trigger cellular senescence or apoptosis (70–72). ROS levels in tumor cells are elevated due to enhanced metabolism, upregulated cell receptor signaling, and increased oxidase activity (73, 74). In response to oxidative stress, tumor cells initiate a self-protective program by upregulating the serine metabolism pathway to produce reducing equivalents, limiting ROS accumulation and supporting cell survival and proliferation (75). During serine metabolism, nicotinamide adenine dinucleotide (NAD^+^) is reduced to nicotinamide adenine dinucleotide (NADH), and serine catabolism via the folate cycle generates GSH, a key intracellular antioxidant (75, 76).

Serine metabolism intersects central carbon and one-carbon pathways, contributing to diverse complex cellular biochemical reactions. The high metabolic activity of tumor cells drives elevated nutrient demand. Serine supplementation promotes tumor cell growth, proliferation, and metastasis. Therefore, given the metabolic dependency of tumor cells on serine, targeted interventions on serine metabolism—such as dietary restriction or pharmacological inhibition of serine synthesis—offer a promising anticancer strategy. Nevertheless, safe and effective therapeutic targeting of serine metabolism remains a major challenge.

## 4 Potential and challenges of serine deprivation therapy

### 4.1 Dietary serine restriction

Serine is essential for tumor growth, prompting the development of novel anticancer therapies that restrict its uptake or metabolism in tumor cells (34, 77). Dietary serine restriction is a direct strategy to reduce its bioavailability to tumor cells. Since serine and glycine are interconvertible, a serine- and glycine-free diet (-SG diet) is typically used to restrict both amino acids simultaneously. This diet markedly inhibits the proliferation of the serine-dependent colorectal cancer (CRC) cell line HCT116 *in vitro* and significantly reduces tumor burden in mouse models of intestinal cancer (Apc inactivation-driven) and lymphomas (Myc activation-driven) *in vivo* (78). Scott et al. further report that an -SG diet inhibits the growth of glioblastoma *in vitro* and *in vivo*, prolonging survival in tumor-bearing mice with good tolerability. Additionally, that serine deprivation synergizes with radiotherapy to enhance therapeutic efficacy *in vivo*. Based on these findings, a clinical trial is being designed to assess its clinical translational potential (79).

In addition to directly suppressing tumors, the serine deficient (Ser Def) diet or -SG diet also enhances antitumor immunity (37, 77, 80, 81). Saha et al. report that serine depletion in CRC cells induces mitochondrial dysfunction, leading to cytosolic accumulation of mitochondrial DNA (mtDNA). Cytosolic mtDNA is detected by cGAS, activating the innate immune cGAS–STING pathway *in vitro* and enhancing antitumor immune infiltration *in vivo*. The Ser Def diet also reprograms the tumor immune microenvironment from an “immunosuppressive” to an “immune-supportive” state, thereby restricting tumor growth (81). The -SG diet enhances antitumor immunity *in vivo* by promoting the infiltration and accumulation of cytotoxic T cells (particularly CD8^+^ T cells) in tumor tissues, thereby suppressing CRC cell proliferation *in vitro* and *in vivo*. However, the -SG diet enhances glycolysis and increases lactate accumulation, leading to programmed death-ligand 1 lactylation and promoting immune evasion. Consequently, combining the -SG diet with anti-PD-1 antibodies effectively blocks this immune evasion and produces synergistic antitumor effects *in vivo* (77). Their single-arm, phase I trial (ChiCTR2300067929) shows that the -SG diet is feasible and safe for modulating systemic immunity, indicating its potential as a combination strategy (77).

Furthermore, SFXN1 mediates serine transport from the cytosol into the mitochondrial matrix and its inhibition mimics serine deprivation at the organelle level, activating a novel innate immune mechanism (59, 60, 82). Li et al. report that SFXN1 knockdown significantly reduced cell proliferation and migration *in vitro*, potentially by inhibiting ERK phosphorylation and CCL20 expression; *in vivo*, targeting SFXN1 decreased Tregs infiltration and suppressed tumor growth (60). Beyond its classical role in one-carbon metabolism, Zhang et al. report a novel pathway through which SFXN1 promotes bladder cancer metastasis through the suppression of PTEN-induced kinase 1 (PINK1)-dependent mitophagy. SFXN1 knockout disrupts this pathway and markedly suppresses proliferation and metastasis *in vitro* and *in vivo* (82). The -SG diet also significantly increases cancer cell sensitivity to radiotherapy *in vitro* and *in vivo* (83). These studies provide strong preclinical evidence for the antitumor efficacy and immunomodulatory mechanisms of serine deprivation, highlighting its therapeutic potential.

**Figure 3:**
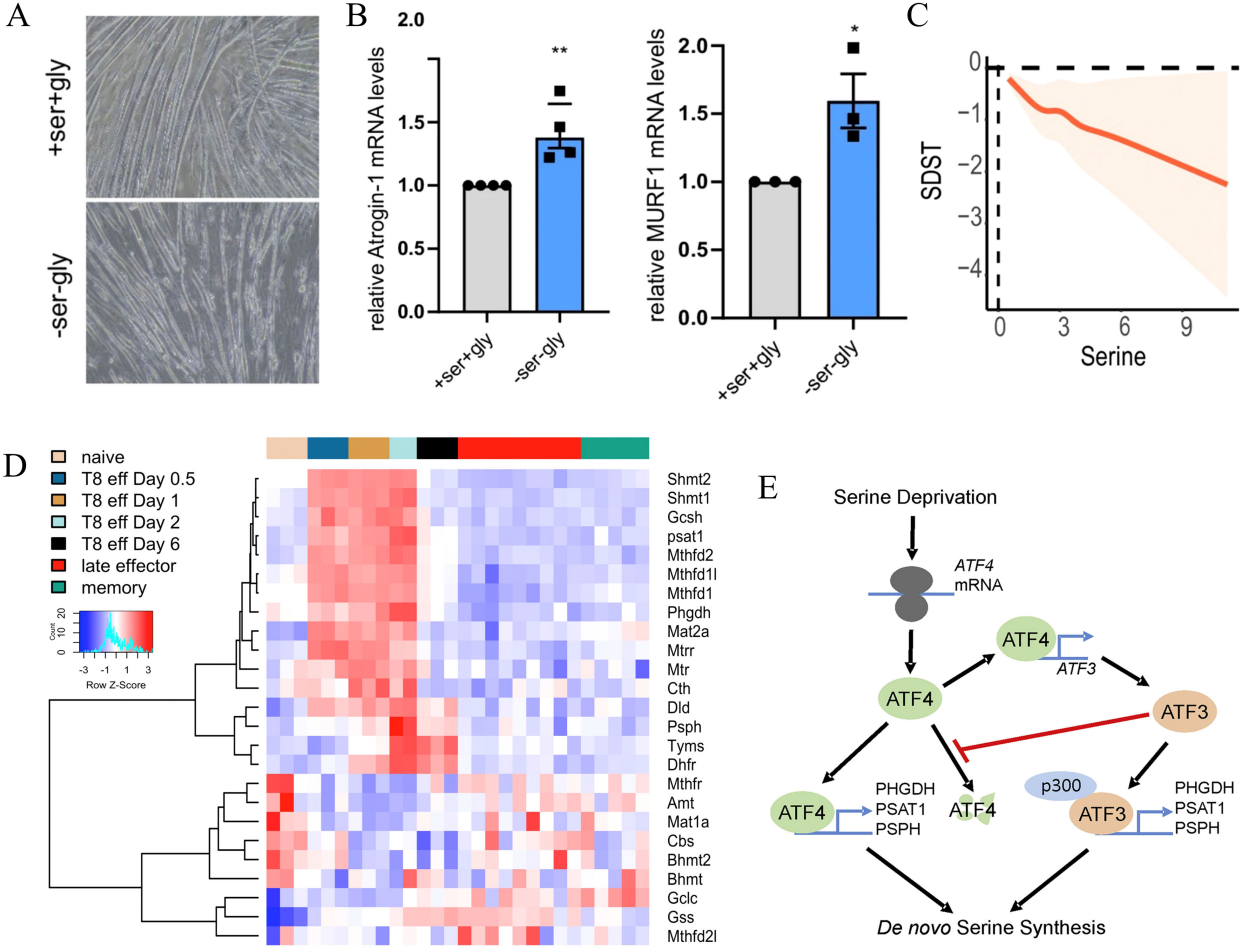
Potential adverse effects of dietary serine restriction on normal tissues. **(A)** Representative microscopic pictures of C2C12 myotubes. Copyright 2024, Springer Nature. **(B)** Dietary serine restriction increases the expression of muscle atrophy-associated genes *Atrogin1* and *MuRF1*. Copyright 2024, Springer Nature. **(C)** SDST results show a downhill-shaped nonlinear relationship between serine intake and cognitive performance (*P* = 0.014). Copyright 2024, Royal Society of Chemistry. **(D)** Temporal expression patterns of SGOC metabolism-related genes in CD8^+^ T cells during the immune response to *LmOVA* infection. The heatmap illustrates relative expression levels of SGOC-related genes across various subsets of CD8^+^ OT-I T cells from the IMMGEN database, including naive, early effector (days 0.5, 1, 2, and 6), late effector (days 8, 10, and 15), and memory (days 45 and 100) T cells. Copyright 2017, Elsevier. **(E)** Dietary serine deprivation activates the SSP and enhances endogenous serine production. Copyright 2021, Cell Press. SDST, Symbol-Digit Substitution Test; SGOC, serine, glycine, one-carbon; SSP, serine synthesis pathway; IMMGEN, Immunological Genome Project.

Nonetheless, implementing dietary serine restriction in clinical settings remains challenging due to various practical limitations. Dietary serine restriction may adversely affect certain normal tissues. Pranzini et al. show that dietary serine deprivation reduces fibers width and inhibits AKT-mTORC1 signaling *in vitro* (**Figure 3A**), resulting in impaired protein synthesis, enhanced protein degradation, and upregulation of the muscle atrophy-associated genes *Atrogin1* and *MuRF1 in vitro* (**Figure 3B**), thereby contributing to cancer-associated weight loss and muscle wasting *in vivo* (84). Epidemiological evidence shows a nonlinear inverse association between dietary serine intake and cognitive performance (P = 0.014; **Figure 3C**), suggesting that serine restriction may impair cognitive function in patients with cancer (85). During early pathogen infection *in vivo*, genes of the serine-glycine-one-carbon (SGOC) metabolic network are coordinately upregulated in CD8+ T cells to support the biosynthetic demands of their rapid proliferation. Among these, *Shmt1* and *Shmt2* are the most strongly upregulated genes (**Figure 3D**) (37). Moreover, the limited clinical efficacy of dietary serine restriction is primarily attributed to the compensatory activation of the endogenous SSP following exogenous serine deprivation (**Figure 3E**) (34, 52). The upregulation of SSP-associated genes promotes CRC cell proliferation and induces resistance to 5-fluorouracil *in vitro* and *in vivo* (38).

While dietary serine restriction shows significant efficacy in preclinical studies, therapeutic strategies targeting this pathway must carefully balance anticancer benefits with the potential adverse effects on normal tissues. Because more than 80% of intracellular serine in tumor cells originates from the SSP, direct inhibition of its key enzymes may offer greater therapeutic efficacy than that of dietary restriction alone. Consequently, dietary serine restriction is more likely to function as an adjuvant or complementary approach within combination therapies.

### 4.2 Targeting key enzymes in the serine synthesis pathway to restrict serine biosynthesis

#### 4.2.1 Inhibition of PHGDH

Emerging evidence demonstrates that the expression levels of key enzymes in the SSP are strongly associated with patient prognosis across multiple cancers. PHGDH, the rate-limiting enzyme in this pathway (86), is overexpressed and drives tumor proliferation, invasion, and therapeutic resistance in various malignancies. In gliomas, PHGDH interacts with the oncogenic transcription factor FOXM1 to promote glioma cell proliferation and invasion (87). In pancreatic cancer, PHGDH expression is significantly upregulated and correlates strongly with tumor size, lymph node metastasis, and clinical stage; furthermore, it serves as an independent prognostic biomarker in affected patients (88, 89). In gastric cancer, PHGDH is significantly overexpressed in multidrug-resistant cells and facilitates resistance development through activation of the PHGDH/IGF2BP1-TCF7L2 axis. Elevated PHGDH expression is strongly associated with poor clinical outcomes in patients with gastric cancer (90). Genetic knockout or site-specific mutation of the PHGDH gene markedly inhibits tumor cell proliferation, underscoring its potential as a therapeutic target in cancer treatment (35, 91). In cervical cancer cells, PHGDH knockout downregulates Bcl-2 expression while upregulating cleaved caspase-3 levels *in vitro*, thereby promoting tumor cell apoptosis *in vivo* (92). Moreover, PHGDH facilitates pancreatic cancer progression *in vivo* by enhancing translation initiation through interactions with eIF4A1 and eIF4E, whereas its downregulation significantly suppresses tumor growth and prolongs overall survival (93).

Recent preclinical studies have evaluated several small-molecule PHGDH inhibitors, demonstrating robust antitumor activity and highlighting their potential as therapeutic agents (94, 95). Spillier et al. report that the anti-alcoholism drug disulfiram inhibits PHGDH enzymatic activity by promoting the conversion of its active tetrameric form into an inactive dimer *in vitro*, thereby exerting significant anticancer effects (96). PHGDH overexpression induces resistance to erlotinib in tumor xenograft mouse models, whereas treatment with the PHGDH inhibitor NCT-503 effectively restores erlotinib sensitivity *in vitro and in vivo* (97). Additionally, compared with monotherapy, the combination of a PHGDH inhibitor with gemcitabine or cisplatin produces synergistic antitumor effects *in vitro* and *in vivo* (98). Beyond its canonical roles, emerging evidence shows noncanonical functions of serine metabolism in driving tumor metastasis. In the cytosol, Soflea et al. demonstrate that purine depletion significant upregulates SSP genes, leading to the accumulation of the intermediate metabolite 3-PS. This metabolite enhances cell migration *in vitro* and *in vivo*, an effect that can be effectively blocked by PHGDH inhibition (99).

Targeting serine metabolism, particularly via PHGDH inhibition, not only suppresses tumor growth through nutrient deprivation but also crucially blocks compensatory pro-metastatic adaptations mediated by the SSP that can be triggered by other therapies, such as chemotherapy. By disrupting these stress-induced adaptive pathways in tumors, PHGDH and SFXN1 inhibitors may offer more comprehensive and durable therapeutic efficacy when combined with conventional treatments. However, despite promising preclinical evidence, no PHGDH inhibitor has yet progressed to clinical trials.

#### 4.2.2 Inhibition of PSATl

Overexpression of PSAT1–the second key enzyme in the SSP–is implicated in the progression of various malignant tumors (100–108). Elevated PSAT1 expression markedly enhances cancer cell proliferation, migration, and invasion, and is strongly correlated with poor patient prognosis in non-small cell lung cancer (NSCLC) (102, 103). Similarly, in breast cancer, PSAT1 overexpression promotes these malignant phenotypes (including enhanced proliferative, migratory, and invasive capacities) and is associated with reduced overall survival (107, 108). Collectively, preclinical studies across multiple cancers demonstrate the therapeutic potential of targeting PSAT1 as an antitumor strategy. Ye et al. demonstrate that PSAT1 knockdown effectively suppresses the proliferation, migration, and invasion of clear cell renal cell carcinoma cells while promoting apoptosis, as confirmed *in vitro* and *in vivo* (104). Furthermore, in esophageal squamous cell carcinoma and lung adenocarcinoma, PSAT1 silencing not only inhibits tumor growth but also acts synergistically with serine deprivation or erlotinib treatment, respectively, to overcome drug resistance *in vitro* and *in vivo* (109, 110).

Although pharmacological inhibition of PSAT1 holds significant promise as an anticancer strategy, the development of PSAT1-specific inhibitors remains in its early stages. Through computational simulations, Zhang et al. predicted that coniferin and tetrahydrocurcumin exhibit strong binding affinities to PSAT1, suggesting their potential as candidate inhibitors. However, these predictions require further validation through *in vitro* and *in vivo* experiments (111). Therefore, future studies should accelerate the identification of PSAT1 inhibitors and their clinical translation by integrating computational technologies (including virtual screening, molecular docking, and simulations) with systematic experimental validation.

#### 4.2.3 Inhibition of PSPH

Inhibition of PSPH, the terminal enzyme in the SSP, effectively suppresses key malignant phenotypes in cancer. PSPH induces autophagy in HCC cells via the AMPK/mTOR/ULK1 signaling pathway, thereby inhibiting apoptosis, while enhancing proliferation and invasion, which collectively accelerate HCC progression (112). Moreover, PSPH suppresses the accumulation of 2-hydroxyglutarate, leading to the activation of proto-oncogene expression and facilitating melanoma growth and metastasis (113). A study also reports that PSPH overexpression in NSCLC tissues is strongly correlated with advanced clinical stages and increased metastatic potential. Knockdown of PSPH significantly suppresses the invasive and migratory abilities of NSCLC cells *in vitro* (114). Currently, PSPH inhibitors remain in the preclinical research phase, with no candidate having advanced clinical trials. Emerging evidence suggests that PSPH knockdown increases tumor cell sensitivity to PD-1 inhibitors. Metformin-mediated PSPH suppression mimics this immunostimulatory effect and acts synergistically with PD-1 targeted therapy (115). Collectively, these findings support PSPH as a promising and druggable therapeutic target in cancer treatment.

In summary, serine deprivation therapy—encompassing dietary restriction and inhibition of the SSP—represents a promising yet underdeveloped strategy in cancer therapeutics. However, its clinical translation has been significantly limited by two major bottlenecks: off-target toxicity and compensatory metabolic adaptations. Therefore, future progress will depend on overcoming these challenges through the development of highly selective inhibitors coupled with precision delivery strategies.

## 5 Nanodelivery systems for serine deprivation

**Figure 4:**
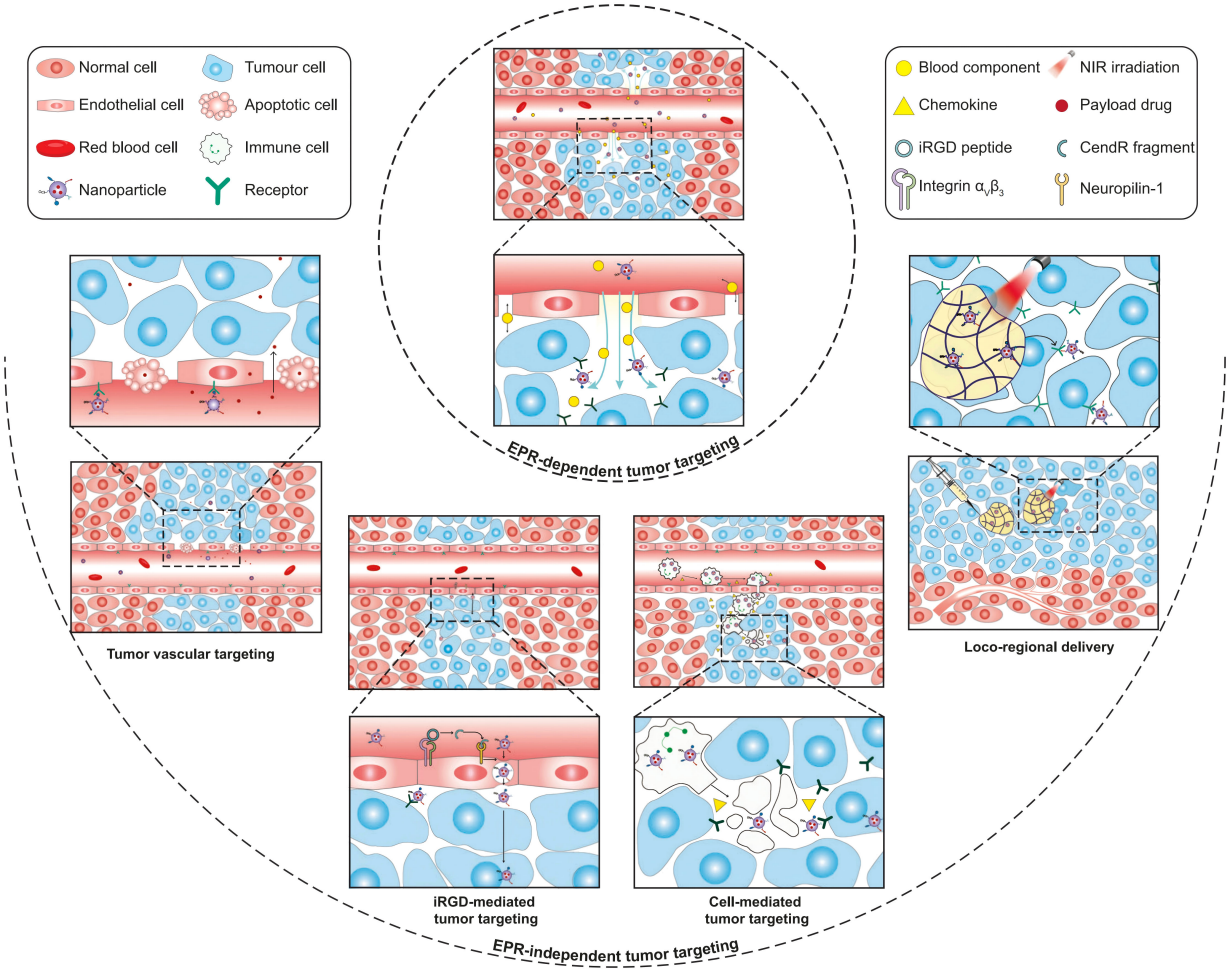
Schematic illustration of EPR-dependent and EPR-independent strategies for nanoparticle delivery to tumors. Copyright 2023, Springer Nature. EPR, enhanced permeability and retention.

Research on SSP inhibition has primarily focused on PHGDH-targeting compounds, including NCT-503 (116), CBR-5884 (117), and WQ-2101 (118). However, these inhibitors generally exhibit poor aqueous solubility and insufficient target specificity, limiting their therapeutic potential. Nanodelivery systems offer a promising strategy to address these pharmacological challenges (119, 120). Encapsulating hydrophobic drugs within nanocarriers not only enhances their solubility and stability but also facilitates tumor-specific delivery through passive targeting (e.g., the enhanced permeability and retention [EPR] effect) (121, 122) and active targeting strategies (e.g., ligand modification) (123–125). To address the substantial heterogeneity of the EPR effect, several EPR-independent tumor-targeting strategies have been proposed, including tumor vascular targeting, cell-mediated tumor delivery, iRGD-mediated tumor targeting, and locoregional administration (125) (**Figure 4**). These approaches collectively enhance therapeutic efficacy while reducing systemic toxicity.

### 5.1 Passive targeting and active targeting strategies of nanodelivery systems

**Figure 5:**
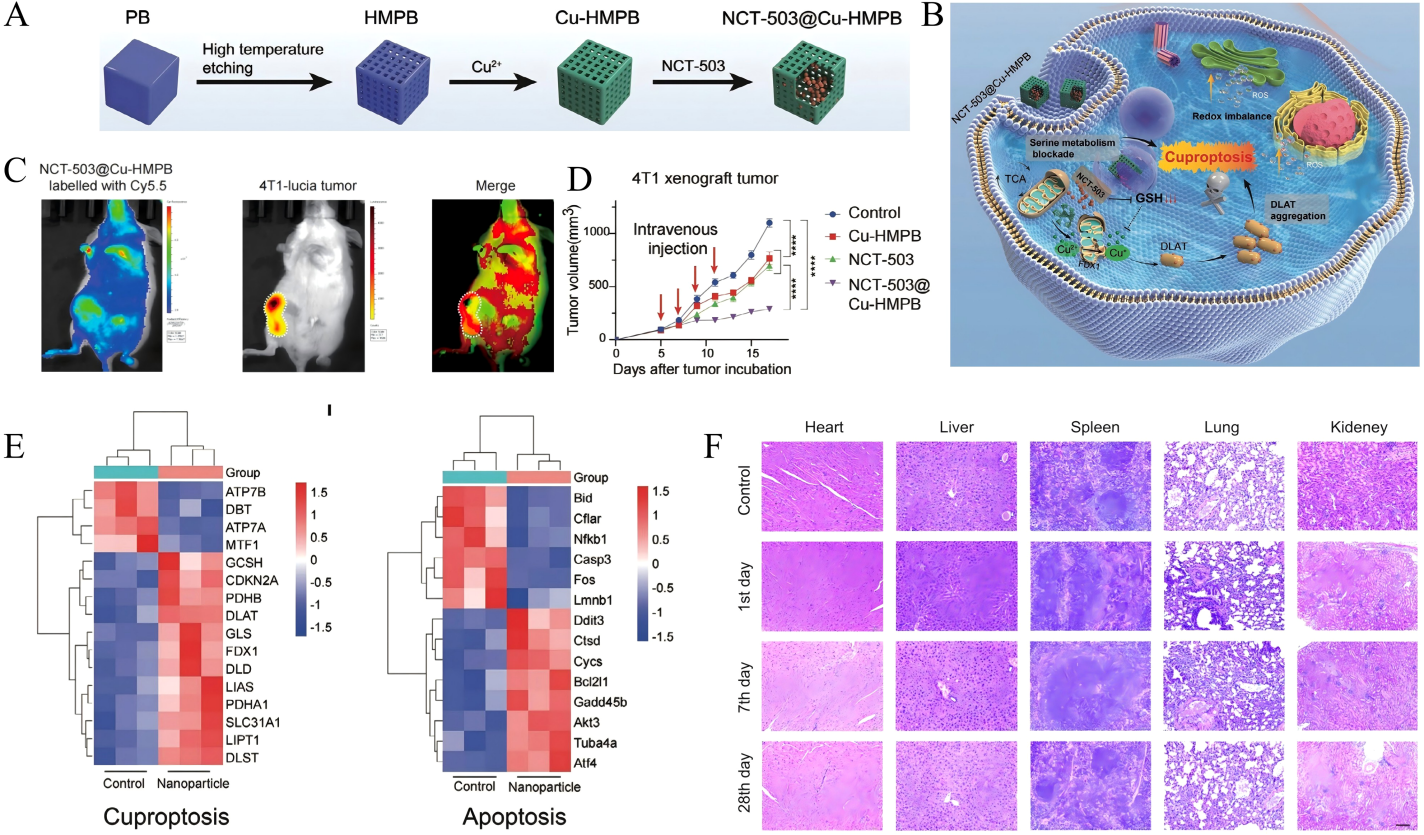
Antitumor effects of NCT-503@Cu-HMPB through induction of cuproptosis and inhibition of serine metabolism. **(A)** Schematic diagram of the synthesis process of NCT-503@Cu-HMPB. **(B)** Schematic diagram of the antitumor mechanism of NCT-503@Cu-HMPB via the induction of cuproptosis and inhibition of serine metabolism. **(C)***In vivo* fluorescence colocalization imaging of Cu-HMPB labeled with Cy5.5 in 4T1-lucia tumor-bearing mice. **(D)** Tumor growth curves of 4T1 xenografts in nude mice. **(E)** Heatmap showing differentially expressed genes involved in the cuproptosis and apoptosis pathways between the nanomedicine-treated and control groups. **(F)** H&E staining of major organs from BALB/c mice treated with NCT-503@Cu-HMPB or untreated, assessed on days 1, 7, and 28. Copyright 2025, Wiley. H&E, hematoxylin and eosin.

At present, research on the nanodelivery systems utilizing passive targeting for the delivery of PHGDH inhibitors in tumor treatment remains limited, though recent studies report preliminary validation. Ma et al. developed a nanoparticle formulation, NCT-503@Cu-HMPB, with **Figure 5A** schematically illustrating its synthesis process. This nanoformulation undergoes acid-triggered degradation within the acidic tumor microenvironment, leading to the simultaneous release of Cu2+ and NCT-503, thereby inducing pH-responsive cuproptosis and inhibiting serine metabolism, as depicted in the mechanistic illustration (**Figure 5B**). *In vivo* fluorescence imaging revealed strong Cy5.5 fluorescence signals localized at the tumor site, demonstrating the effective tumor-targeted accumulation of Cu-HMPB mediated by the EPR effect (**Figure 5C**). Consequently, treatment with this nanoparticle markedly suppresses tumor growth (**Figure 5D**) without inducing evident toxicity in major normal organs *in vivo* (**Figure 5F**) (126). Additionally, transcriptomic analysis *in vivo* (**Figure 5E**) confirms that NCT-503@Cu-HMPB simultaneously activates cuproptosis and apoptosis pathways while inhibiting serine synthesis-mediated proliferation, highlighting its potential as a dual-action anticancer therapy (126).

Currently, research on active targeting-based nanodelivery of PHGDH inhibitors for tumor treatment remains limited. Nevertheless, active targeting strategies developed for other therapeutic agents provide valuable insights for designing future PHGDH inhibitor delivery systems (127, 128). For instance, Han et al. conjugated cRGD peptides to the surface of Ag_2_S nanoparticles through amide bonding, thereby enhancing the tumor-targeting capability of the nanocarrier, significantly improving antitumor efficacy, and minimizing off-target toxicity in nontumor tissues *in vivo* (129). Similarly, Ren et al. developed nanoassemblies named MC@RL/Apt, composed of a red blood cell-liposome hybrid membrane-camouflaged Mn–Ce6 nanocomplex. *In vitro* studies show that MC@RL/Apt functions as an efferocytosis inhibitor by suppressing macrophage phagocytosis of apoptotic cells and promoting macrophage polarization toward the pro-inflammatory M1 phenotype. Consequently, treatment with MC@RL/Apt effectively suppresses tumor growth and elicits robust antitumor immune responses *in vivo* (130). This study not only highlights the advantages of nanocarriers in drug protection and targeted delivery but also demonstrates that active targeting strategies can precisely modulate the TIME. Collectively, these findings validate the efficacy of passive and active targeting approaches in nanoscale delivery systems *in vitro* and *in vivo*, providing valuable guidance for the rational design of nanocarriers to deliver PHGDH inhibitors.

### 5.2 Nano-based co-delivery system

However, nanomaterial-based delivery strategies for PHGDH inhibitors face challenges in completely depleting all sources of serine within tumors, as tumor cells can still import exogenous serine to partially sustain their growth (33). Montrose et al. report that in colon cancer models, combining the Ser Def diet (which restricts exogenous supply) with PSAT1 knockout (which blocks endogenous synthesis) results in greater tumor growth suppression *in vitro* and *in vivo* than that of either intervention alone (105). This finding indicates that the concurrent inhibition of exogenous and endogenous serine sources in tumor cells significantly enhances antitumor efficacy, offering a promising therapeutic strategy (105, 131). GPNA and V-9302 act as competitive inhibitors of the ASCT2 transporter by binding to its substrate-binding site, thereby blocking serine uptake and depleting tumor cells of exogenous serine (132).

While the co-delivery of PHGDH and ASCT2 inhibitors remains unreported, studies demonstrate the potential of nanocarrier-based co-delivery systems to achieve synergistic therapeutic effects through the simultaneous transport of multiple agents. For example, Yu et al. developed a polymeric nanosystem for the co-delivery of resiquimod (R848) and rifapentine, which synergistically reprograms macrophages and enhances intracellular antibacterial activity in bacterial infection models, achieving significantly higher efficacy than that of monotherapy *in vitro* and *in vivo* (133). Similarly, Sun et al. constructed a cyclodextrin-based nanoparticle for the co-delivery of ginsenoside Rg3 and quercetin, which effectively suppresses tumor growth and remodels the tumor microenvironment, demonstrating strong chemo-immunotherapeutic synergy in a CRC model *in vitro* and *in vivo* (134). Collectively, these studies establish a solid technical foundation for developing nanocarrier-based co-delivery strategies. Building on this foundation, the co-delivery of PHGDH and ASCT2 inhibitors using nanodelivery systems may enable the comprehensive blockade of exogenous and endogenous serine sources within tumors while leveraging nanosystem-targeting strategies to promote drug accumulation at tumor sites. This strategy is expected to enhance antitumor efficacy, and reduce adverse effects on nontumor tissues.

In summary, nanosystem-based strategies encompassing targeted delivery and co-delivery approaches hold considerable potential to improve the therapeutic efficacy of PHGDH inhibitors. However, research specifically addressing the nanodelivery of PHGDH inhibitors remains limited, as most existing studies focus on nanodelivery systems for other therapeutic agents. The feasibility, synergistic interactions, and safety profiles of such approaches require further extensive preclinical validation. Therefore, continued in-depth research is warranted to overcome these challenges and accelerate clinical advancement.

## 6 Summary and outlook

In conclusion, the metabolic reliance of tumor cells on serine metabolism constitutes a key therapeutic vulnerability for targeted cancer treatment. The SSP is primarily regulated by three rate-limiting enzymes: PHGDH, PSAT1, and PSPH. Current research predominantly focuses on developing PHGDH inhibitors; however, no PHGDH inhibitor has received clinical approval from the FDA. In contrast, research on inhibitors targeting PSAT1 and PSPH remains underdeveloped, with only limited progress in drug discovery efforts. The off-target distribution of PHGDH inhibitors may induce adverse effects in nontumor tissues, but nanoparticle-based delivery systems offer a promising strategy to address this challenge. The co-delivery of PHGDH and ASCT2 inhibitors using nanoparticle-based delivery systems represents a highly promising direction for future cancer therapy research. However, the feasibility, synergistic efficacy, and safety profile of this strategy require systematic preclinical validation. Therefore, future studies should focus on developing selective inhibitors targeting PHGDH, PSAT1, PSPH, and ASCT2, while optimizing nanoparticle-based delivery systems to enhance delivery efficiency. In addition, further exploration of combination therapeutic strategies is warranted to facilitate the clinical translation and broader application of serine metabolism-targeted therapies.
